# Chemotherapeutic Stress Induces Transdifferentiation of Glioblastoma Cells to Endothelial Cells and Promotes Vascular Mimicry

**DOI:** 10.1155/2019/6107456

**Published:** 2019-06-18

**Authors:** Shivani Baisiwala, Brenda Auffinger, Seamus P. Caragher, Jack M. Shireman, Riasat Ahsan, Gina Lee, Tanwir Hasan, Cheol Park, Miranda R. Saathoff, Anne C. Christensen, Atique U. Ahmed

**Affiliations:** ^1^Department of Neurological Surgery, Feinberg School of Medicine, Northwestern University, Chicago, IL, USA; ^2^Winona State University, Winona, MN, USA

## Abstract

Glioblastoma (GBM) is the most common and aggressive primary malignant brain tumor affecting adults, with a median survival of approximately 21 months. One key factor underlying the limited efficacy of current treatment modalities is the remarkable plasticity exhibited by GBM cells, which allows them to effectively adapt to changes induced by anticancer therapeutics. Moreover, GBM tumors are highly vascularized with aberrant vessels that complicate the delivery of antitumor agents. Recent research has demonstrated that GBM cells have the ability to transdifferentiate into endothelial cells (ECs), illustrating that GBM cells may use plasticity in concert with vascularization leading to the creation of tumor-derived blood vessels. The mechanism behind this transdifferentiation, however, remains unclear. Here, we show that treatment with temozolomide (TMZ) chemotherapy induces time-dependent expression of markers for glioma stem cells (GSCs) and immature and mature ECs. In addition, GBM tumors growing as orthotopic xenografts in nude mice showed increased expression of GSC and EC markers after TMZ treatment. *Ex vivo* FACS analysis showed the presence of immature and mature EC populations. Furthermore, immunofluorescence analysis revealed increased tumor-derived vessels in TMZ-recurrent tumors. Overall, this study identifies chemotherapeutic stress as a new driver of transdifferentiation of tumor cells to endothelial cells and highlights cellular plasticity as a key player in therapeutic resistance and tumor recurrence.

## 1. Introduction

Both standard and novel therapies for glioblastoma (GBM), the most aggressive primary adult brain tumor, have largely failed to improve outcomes for patients. While the current standard of care—surgical resection followed by radiation and temozolomide (TMZ) chemotherapy—initially reduces tumor volume, therapy-resistant tumors invariably develop [1]. One factor known to contribute to GBM's intractability is the close relationship between GBM tumor cells and surrounding blood vessels [2]. GBM is a highly vascularized cancer and contains excessive levels of vascular endothelial growth factor (VEGF) [3, 4]. Blood vessels provide a protumorigenic environment, closely intertwine with the tumor cells, and enhance many critical aspects of their survivorship including maintaining the niche necessary for therapy-resistant glioma stem cells as well as promoting the invasive capacity of these tumor cells [5–8]. Recent work has shown that, in addition to liaising with surrounding blood vessels, GBM tumors can generate their own vessels in a process termed vascular mimicry where GBM cells transdifferentiate into functional endothelial cells [9–13]. Critically, the extent of vascular mimicry negatively correlates with patient prognosis [14]. However, the influence of standard of care therapy on vascular mimicry remains poorly understood.

Our group and others have previously shown that therapeutic stress can activate cellular plasticity in GBM, leading to enrichment of the glioma stem-like cell (GSC) population. GSCs are characterized by heightened self-renewal capacity and elevated resistance to therapy [15–20]. Critically, our work demonstrated that this conversion utilizes hypoxia-inducible factors (HIF), which are stabilized during chemotherapeutic stress [21]. Hypoxia canonically leads to activation of angiogenesis [22], and current evidence suggests that a similar process may drive vascular mimicry [23, 24]. Because of this, we set out to examine how therapeutic stress influences transdifferentiation and vascular mimicry. Using a mix of patient-derived xenograft cell lines and murine models, we analyzed how treatment with TMZ, the most commonly used antiglioma chemotherapy, influences the expression of GSC and endothelial cell (EC) markers. This analysis revealed that chemotherapy induces the formation of a subpopulation of tumor cells expressing both GSC and intermediate EC markers as well as another population expressing GSC and mature EC markers. Tube formation assays and analysis of orthotopic xenografts confirmed that therapy increases transdifferentiation and the formation of tumor-derived vessels.

## 2. Materials and Methods

### 2.1. Cell Lines and Culture

U251 and A172 human glioma cell lines were procured from the American Type Culture Collection (Manassas, VA, USA). These cells were cultured in Dulbecco's Modified Eagle's Medium (DMEM; HyClone, Thermo Fisher Scientific, San Jose, CA, USA) supplemented with 10% fetal bovine serum (FBS; Atlanta Biologicals, Lawrenceville, GA, USA) and 2% penicillin-streptomycin antibiotic mixture (Cellgro, Herndon, VA, USA; Mediatech, Herndon, VA, USA).

Patient-derived xenograft (PDX) glioma specimens (GBM43, GBM12, and GBM6) were obtained from Dr. C. David James at Northwestern University and maintained according to published protocols [25].

### 2.2. Animals

Athymic nude mice (nu/nu; Charles River, Skokie, IL, USA) were housed according to all Institutional Animal Care and Use Committee (IACUC) guidelines and in compliance with all applicable federal and state statutes governing the use of animals for biomedical research. Briefly, animals were housed in shoebox cages. Food and water were available ad libitum. A strict 12-hour light-dark cycle was maintained.

Intracranial implantation of glioblastoma cells was performed as previously published [21]. Briefly, animals received prophylactic injection of buprenex and metacam via intraperitoneal (i.p.) injection, followed by an i.p. injection of ketamine/xylazine anesthesia mixture (Henry Schein; New York, NY, USA). Sedation was confirmed by foot pinch. Artificial tears were applied to each eye, and the scalp was sterilized repeatedly with betadine and ethanol. The scalp was then bisected using a scalpel to expose the skull. A drill was used to make a small burr hole above the right frontal lobe (approximately 1 mm in diameter). The animals were then placed into a stereotactic rig, and a Hamilton syringe loaded with the cells was brought into the burr hole. The needle point was lowered 3 mm from the dura, and injection of 5 *μ*L of cell mixture took place over one minute. The needle was then raised slightly and left undisturbed for 1 minute to ensure proper release of the cell mixture. After this minute, the syringe was carefully removed. The animal's head position was maintained, and the skin of the scalp was closed with sutures (Ethicon; Cincinnati, OH, USA).

Drug treatments were initiated seven days after intracranial implantation. Animals received i.p. injections of either TMZ (2.5 mg/kg-10 mg/kg) or equimolar DMSO. Injections were performed daily for five consecutive days.

For *ex vivo* FACS experiments, all mice were sacrificed 5 days after the last injection of TMZ. For recurrent xenograft model experiments, 3 groups in total were created: one control group, one primary group, and one recurrent group. All groups followed the same treatment plans; however, the primary group animals were euthanized 3 days following the cessation of TMZ treatment for analysis of cells that were exposed to but had not yet developed resistance to TMZ. Control and recurrent animals were monitored daily for signs of sickness, including reduction in body weight, lowered body temperature, lack of grooming, hunched appearance, and behavioral signs by a blinded experimenter. Animals were euthanized when, in accordance with IACUC protocols and at the recommendation of animal health care technicians or staff veterinarians, it was determined that they would not survive until the next day. Animal sacrifices were performed according to Northwestern University guidelines. For those brains utilized for FACS analysis, please see Flow Cytometry in Materials and Methods.

### 2.3. Flow Cytometry

For *in vitro* experiments, cells were collected at serial time points after the beginning of treatment with TMZ or DMSO (days 2, 4, 6, and 8), and fresh surface staining was performed. Live cells were collected and rinsed in PBS. For uptake experiments, cells were then incubated with appropriate antibodies. After 30-minute incubation at 4°C, cells were washed with PBS thoroughly. Preincubated QDOTs were then added to each well and allowed to incubate at room temperature for 4 minutes. After a final PBS wash, cells were analyzed using the flow cytometer.

*In vivo* studies began with the sacrifice of tumor-bearing mice and immediate removal of the whole brain. Brains were washed in ice-cold PBS and then bisected down the longitudinal fissure, and the right brains (tumor-bearing) were passed through a 70 *μ*M strainer. These single cell suspensions were then incubated in ACK lysis buffer (Lonza; Walkersville, MA, USA) for 5 minutes at 20-25°C to lysis any blood cells. After washing with PBS, cells were stained as in the *in vitro* experiments. Human leukocyte antigen (HLA) staining was used to identify human tumor cells. All cells were collected in PBS supplemented with 1% bovine serum albumin (BSA; Fisher Scientific, Fair Lawn, NJ, USA) and sodium azide and kept on ice until read.

### 2.4. Immunofluorescence Staining

Following euthanasia, mice were perfused with ice-cold PBS. The brains were then frozen in cryoprotectant on dry ice and subsequently stored at -80°C. Samples were sectioned (8 *μ*m) and then stained according to previously described protocols [26]. Briefly, sections were thawed for 30 minutes at room temperature. They were then washed with PBS 3 times for 5 minutes to remove any remaining cryoprotectant. Sections were fixed in 4% PFA at room temperature for 15 minutes and were subsequently washed 3 times in PBS for 5 minutes each. Next, sections were blocked and permeabilized in a 10% BSA solution with Triton-X (Thermo Fisher Scientific) for 1.5 hours at room temperature. Sections were incubated overnight at 4 degrees with primary antibodies diluted in 1% BSA+Triton-X (Thermo Fisher Scientific). The next morning, sections were washed 3 times for 10 minutes each in PBS. Following these washes, secondary antibodies diluted in 1% BSA+Triton-X were added (Thermo Fisher Scientific). Sections were incubated in the secondary antibodies for 2.5 hours at room temperature and were then washed in PBS 3 times for 10 minutes each. Finally, samples were mounted with a DAPI-containing mounting media (Invitrogen) and imaged by fluorescence microscopy (Leica). Images were compiled and analyzed in ImageJ.

### 2.5. LDL Uptake Assay

GBM cells treated with either DMSO or TMZ and following treatment were incubated with fluorescently labeled LDL protein and incubated for 4 hours. After incubation, cells were prepared for FACS according to standard protocols. Measurement of fluorescence uptake was then recorded and different cellular populations were analyzed.

### 2.6. Tube-Forming Assay

GBM cells were cultured as appropriate. For more information, please see Cell Lines and Culture. Experimental GBM cells were treated for 8 days with DMSO or TMZ in EBM, EGM, or DMEM media. (EBM and EGM media are optimized for growth of endothelial cells). On day 8 of treatment, 100 *μ*L of freshly thawed Matrigel was used to coat the wells of a 96-well plate in order to create a gel substrate. The experimental GBM cells were then plated into the wells at low densities. Cells were incubated at 37 degrees for 16 hours and were subsequently imaged by a blinded experimenter. The number of tubes per field was counted for each well to determine the tube-forming capacity of the cells.

### 2.7. Statistical Analysis

All statistical analyses were performed using the GraphPad Prism Software v4.0 (GraphPad Software; San Diego, CA, USA). In general, data were presented as mean (SD) for continuous variables and number (percentage) for categorical variables. Differences between two groups were assessed using Student's *t*-test or the Wilcoxon rank sum test as appropriate. Difference among multiple groups was evaluated using analysis of variance (ANOVA) with Tukey's post hoc test or Mann-Whitney *U* test followed by Bonferroni correction as appropriate. Survival curves will be graphed via the Kaplan-Meier method and compared by log-rank test. All tests were two-sided and a *p* value < 0.05 was considered statistically significant.

## 3. Results and Discussion

### 3.1. TMZ Treatment Alters the Expression of Markers for Glioma Stem-Like Cells and Both Intermediate and Mature Endothelial Cells

**Figure 1:**
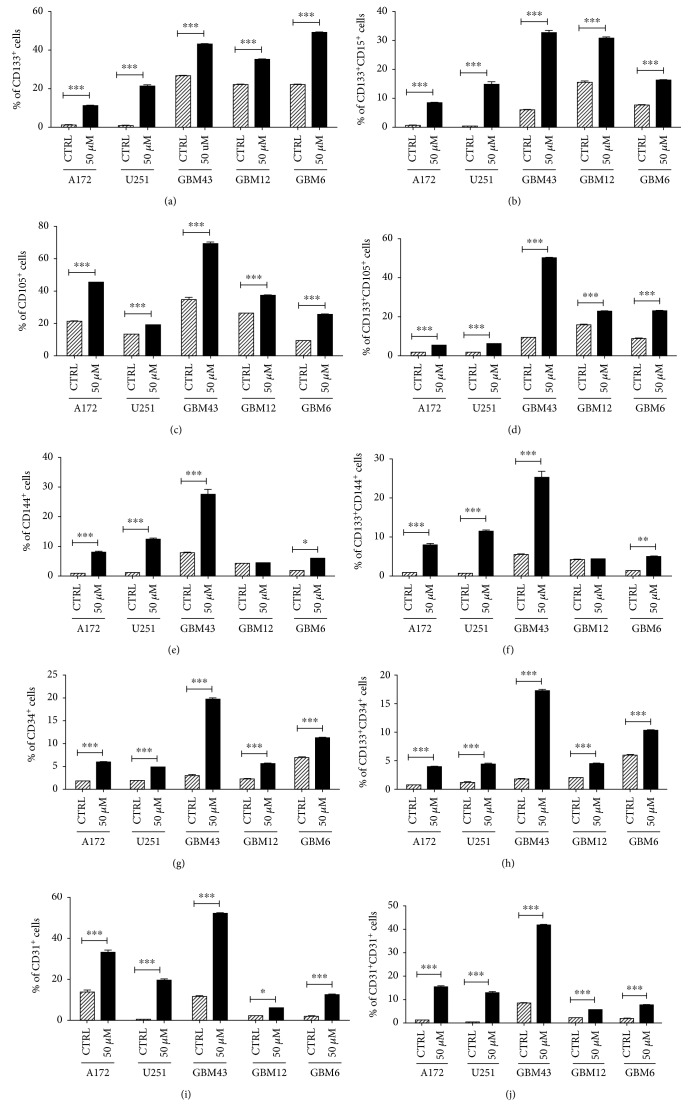
TMZ treatment induces expression of endothelial makers in patient-derived GBMs. A panel of human glioma cell lines (A172 and U251) and patient-derived xenograft cells (GBM43, GBM12, and GBM6) was treated with TMZ (50 *μ*M) or equimolar DMSO (CTRL). After 8 days, cells were analyzed by flow cytometry for the percentage of cells positive for (a) CD133, a maker of glioma stem cells (GSC); (c) CD105 and (e) CD144, markers of intermediate endothelial cells; and (d) CD34 and (e) CD31, markers of mature endothelial cells. To confirm the induction of a GSC population, cells were analyzed for CD133 and CD15. In addition, the population of cells positive for both CD133 and each endothelial marker was analyzed (d, f, h, j). Bars represent the means of three independent experiments, and error bars show the standard error measure. The effect of TMZ was compared in each cell line by the Student *t*-test with Tukey's post hoc test.

To begin our investigation of potential connections between therapeutic stress and transdifferentiation, we performed flow cytometry analysis on a panel of human GBM cells treated with a physiological dose of TMZ (50 *μ*M) or equimolar DMSO [27–30]. After 8 days, expression of the GSC markers CD133 and CD15 was assayed, as well as levels of CD105 and CD144, intermediate endothelial cell (EC) markers, and CD34 and CD31, markers of mature ECs (Figure 1) [31–33]. In all three PDX lines and GBM cell lines tested, TMZ increased CD133 expressing cells, consistent with our previous studies [21, 26], as well as the percentage of cells CD133^+^CD15^+^ (Figures 1(a) and 1(b)). Basal expression of EC markers varied across different cell lines, reflecting the well-established heterogeneity of GBM [34, 35]. TMZ treatment induced the expression of EC markers in all cell lines, except CD144 expression in GBM12. Critically, examination of the subpopulation of GBM cells positive for both CD133 and each EC marker revealed the presence of a GSC^+^EC^+^ population following TMZ exposure (Figures 1(c)–1(j)). Clearly chemotherapeutic stress influences the expression of EC markers, potentially generating a population of GSCs en route to obtaining an EC phenotype.

**Figure 2:**
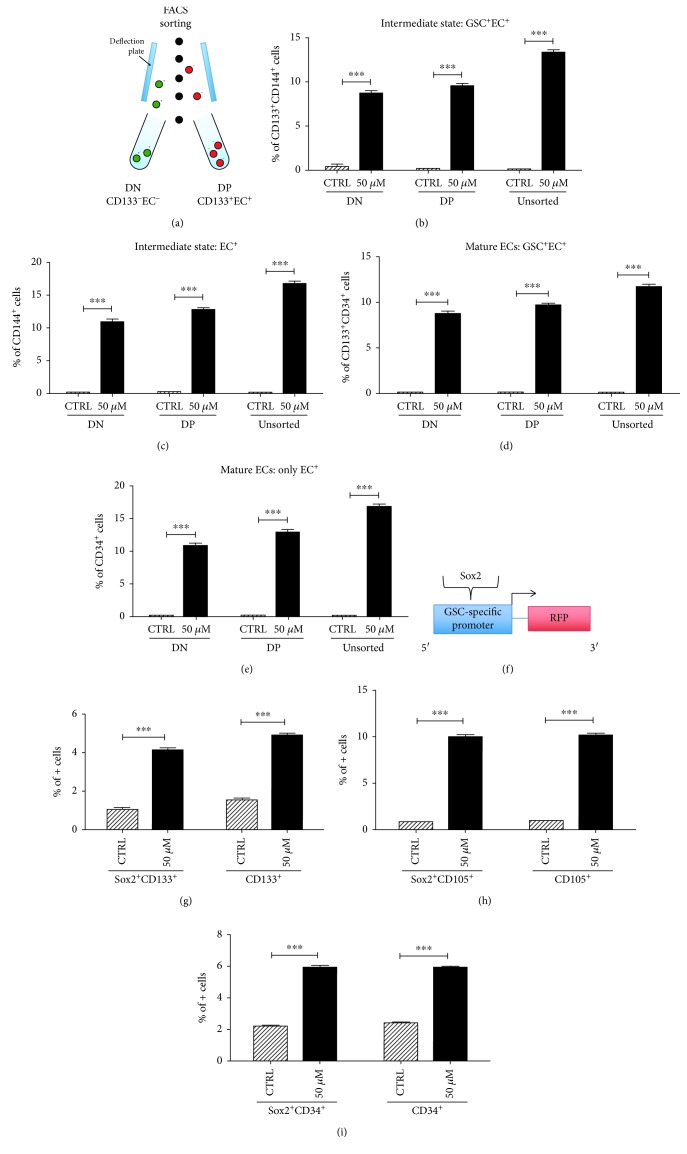
TMZ treatment induces expression of endothelial markers in both GSC^+^/EC^+^ and GSC^−^/EC^−^ populations. The PDX line GBM43 was treated with 50 *μ*M TMZ or equimolar DMSO after being divided into 3 populations—EC^+^/GSC^+^, EC^−^/GSC^−^, or unsorted (a). After 8 days, cells were analyzed by flow cytometry for the percentage of cells positive for (b) CD133/CD144, reflecting cells transitioning from a stem state to an immature EC state; (c) CD144, reflecting cells in an immature EC state; (d) CD133/CD34, reflecting cells transitioning from a stem state to a mature EC state; and (e) CD34, reflecting cells in a mature EC state. To confirm the results, a promoter-reporter system that is highly specific for Sox2 expression (another GSC marker) was also used (f). After treatment for 8 days, flow cytometry was used once more to determine the population of cells positive for (g) Sox2/CD133 and CD133, reflecting the GSC state; (h) Sox2/CD105 and CD105, reflecting the transition to immature ECs and the immature EC state, respectively; and (i) Sox2/CD34 and CD34, reflecting the transition to mature ECs and the mature EC state, respectively. Bars represent the means of three independent experiments, and error bars show the standard error measure. The effect of TMZ was compared in each population by the Student *t*-test with Tukey's post hoc test.

Our next step was to assess how therapeutic stress affected specific subpopulations of cells. We performed flow cytometry sorting on a panel of human GBM cells. Cells were isolated based on whether they were positive or negative for the stem surface marker CD133 and whether they were positive or negative for EC markers CD31 and CD34. Three populations of cells were isolated—double negative (CD133^−^, EC^−^), double positive (CD133^+^, EC^+^), and unsorted (Figure 2(a)). Each population was treated for 8 days with 50 *μ*M TMZ or equimolar DMSO. After 8 days, flow cytometry was used to analyze the expression of CD133 (a stem cell maker), CD144 (an intermediate EC marker), and CD34 (a mature EC marker). In each population, TMZ increased the CD133/CD144 expression as well as the total CD144 expression (Figures 2(b) and 2(c)). This suggests an upregulation in the number of cells transitioning from stem cells to ECs as well as an upregulation in ECs. Furthermore, in each population, TMZ increased the CD133/CD34 expression as well as the total CD34 expression, suggesting a concomitant increase in the number of cells transitioning to a mature EC state and an upregulation in mature ECs (Figures 2(c) and 2(d)). The change seen across all three populations reflects the high level of plasticity that has been previously described in GBM. Even the double negative population, when exposed to therapeutic stress, is able to dedifferentiate to a stem-like state and then redifferentiate down an endothelial cell pathway.

To further investigate this plasticity, we developed a promoter-reporter system that identifies GSCs with high specificity [21]. A Sox2 promoter was linked to an RFP reporter, and human GBM cell lines were transfected with the plasmid (Figure 2(e)). Cells were treated with 50 *μ*M TMZ or equimolar DMSO for 8 days. Flow cytometry showed that the population of Sox2^+^/CD133^+^ cells and the population of CD133^+^ cells were significantly increased after therapeutic stress, suggesting that therapeutic stress does indeed promote stemness and further showing the plasticity of GBM cells. Similar methods were used to assess the level of CD105/Sox2 coexpression and CD34/Sox2 coexpression, confirming that the number of immature ECs and mature ECs is also upregulated after therapeutic stress (Figures 2(f) and 2(g)). Overall, these data show that therapeutic stress alters the expression of markers and promotes GSCs and differentiation down an EC pathway.

### 3.2. Functional Validation of Therapeutic Stress-Induced EC

**Figure 3:**
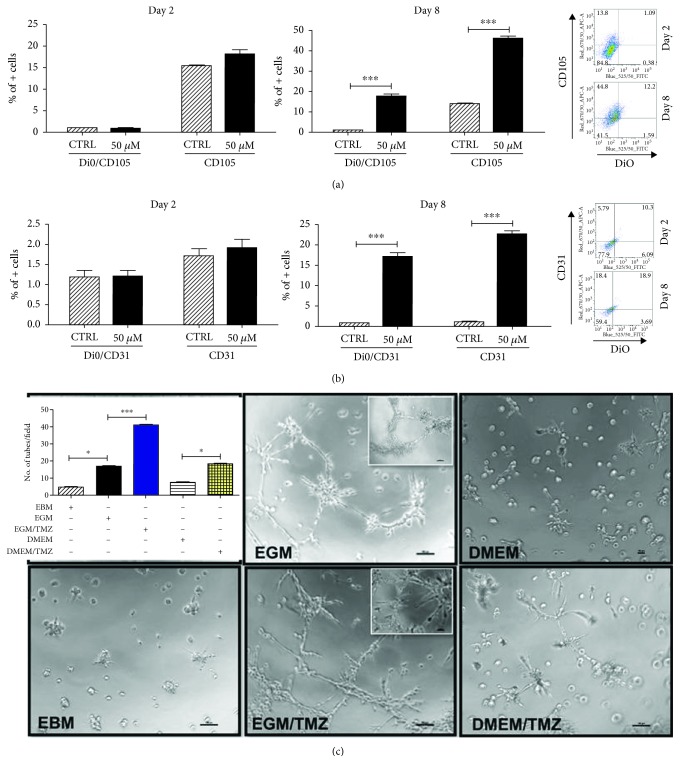
TMZ increases population of immature and mature endothelial cells with functional endothelial cell properties. A Dil-Ac-LDL uptake assay was performed, where acetylated (Ac), fluorescently labeled low-density lipoprotein (LDL) specifically binds to a receptor on the surface of the endothelial cells delivering cholesterol via endocytosis. (a) FACS analysis was performed at day 2 and day 8 post-TMZ exposure to evaluate the uptake of Dil-Ac-LDL by the CD105- (a) and CD31- (b) positive cells. (c) The PDX line GBM43 was treated with 50 *μ*M TMZ or equimolar DMSO and then plated in EGM (optimized endothelial cell media with 2% FBS and VEGF for rapid proliferation), EBM (EGM media without any supplement and growth factors), or DMEM media. Results show that EGM media independently promoted tube formation which was significantly augmented in the presence of TMZ. In addition, while DMEM media alone did not promote tube formation, the addition of therapeutic stress significantly increased tube formation. Images were taken and analyzed by a blinded experimenter. Bars represent the means of three independent experiments, and error bars show the standard error measure. Tubes were quantified based on the number of tubes per field with images taken across the plate of cells. The effect of various treatments was compared across cells by the Student *t*-test with Tukey's post hoc test.

While expression of EC markers is interesting, it does not confirm that chemotherapeutic stress is actually inducing the formation of functional vessels capable of ferrying blood to the tumor. In order to examine the functional effects of TMZ on GBM cells, we first performed a Dil-Ac-LDL uptake assay, where acetylated (Ac), fluorescently labeled low-density lipoprotein (LDL) that specifically binds to a receptor on the surface of the endothelial cells delivering cholesterol via endocytosis was used to evaluate the functionality of the therapeutic stress-induced endothelial-like GBM cells. PDX cells GBM43 were cultured in media containing TMZ in the presence of Dil-Ac-LDL. FACS analysis was performed at day 2 and day 8 post-TMZ exposure to evaluate the uptake of Dil-Ac-LDL by the CD105- (Figure 3(a)) and CD31- (Figure 3(b)) positive cells. Next, we performed a tube-formation assay, where PDX GBM cells were plated in the presence of different media types (EGM, EBM, and DMEM) and treated with either DMSO or TMZ (50 *μ*Μ) for 8 days. They were then transferred to Matrigel and grown overnight. Live cells were imaged by a blinded experimenter and tubes were counted (Figure 3). EGM induced formation of tubes, as expected; this increase was further activated by treatment with TMZ. However, cells growing in DMEM (which is not a tube-activating media) showed significant tube formation when exposed to TMZ as well. These results, combined with the previously described FACS data, strongly suggest that chemotherapy induces GBM cells to form blood vessels, independent of other factors.

### 3.3. Orthotopic Xenografts Treated with TMZ Exhibit Increased EC Marker Expression in Tumor Cells

**Figure 4:**
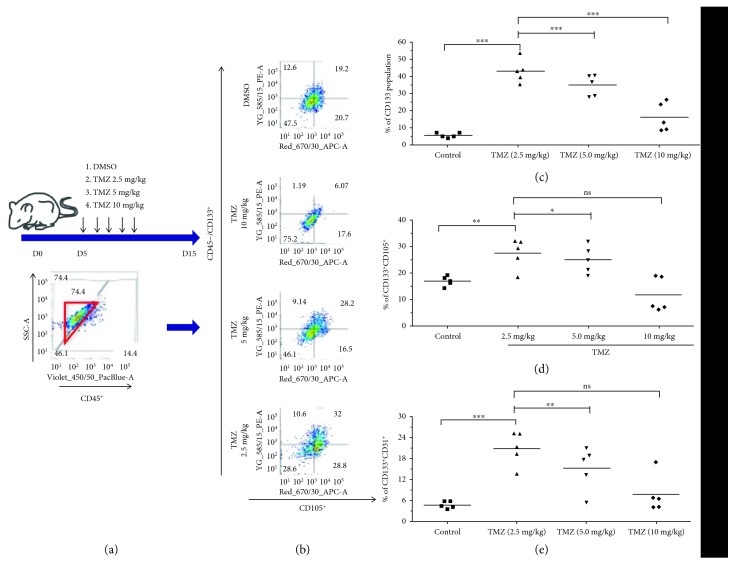
Orthotopic xenograft tumors treated with TMZ exhibit elevated expression of markers of both immature and mature endothelial cells. (a) Athymic nude mice had PDX GBM cells implanted intracranially in the right cerebral hemisphere. Five days later, treatment was initiated, with three groups of mice receiving different doses of TMZ (2.5, 5, or 10 mg/kg, i.p.). Mice treated with DMSO served as a control (*n* = 5, in both male and female mice). Mice were given the drug each day for five consecutive days. Five days after cessation of treatment, mice were sacrificed and brains collected for ex vivo flow cytometry analysis. The inset flow cytometry plot shows how human PDX GBM cells were identified and how mouse cells were excluded, based on CD45 staining. (b) Representative FACS plots from each treatment group showing the percentages of cells positive for CD133, a GSC marker, and CD105, an intermediate endothelial cell marker. (c) Quantification of the CD133^+^ population in each treatment group. (d, e) Quantification of the percentage of human tumor cells positive for both CD133 and (d) CD105 and (e) CD31, markers of endothelial cells. Dots represent means from individual animals. The means of all mice were compared by one-way ANOVA with multiple comparisons. ^∗^ *p* < .05, ^∗∗^ *p* < .01, ^∗∗∗^ *p* < .001. *N* = 5 mice/group.

Thus far, all described experiments have been performed *in vitro*. To determine if these effects are actually observed when cells are growing *in vivo*, PDX GBM cells were implanted in the brains of athymic nude mice (150,000 cells/mouse). Five days later, animals were treated with TMZ at doses of 2.5, 5, or 10 mg/kg body weight; control animals were treated with equal volumes of DMSO. Treatments occurred daily for a total of five days. Five days after completion of treatment, mice were sacrificed and *ex vivo* flow cytometry was performed. CD45 status was used to exclude mouse cells from analysis. Interestingly, we observed an inverse relationship between dose of chemotherapy and expression of CD133 alone. An inverse relationship was also observed between TMZ dose and percentage of cells positive for both CD133 and EC markers (Figure 4). This was true for both CD105, a marker of intermediate ECs, and CD31, a marker of mature endothelial cells. Further exploration showed that this inverse relationship was also demonstrated in vitro across a range of dosing from 50 to 300 micromolar TMZ with the exception of CD133 and CD31 double positive cells at day 4 (Figure S2). These results confirm that therapeutic stress induces the expression of EC markers in GBM and suggest that expression of these markers is dynamic and could be dose and time dependent.

### 3.4. Recurrent Tumors Contain Elevated Numbers of Tumor-Derived Vessels

**Figure 5:**
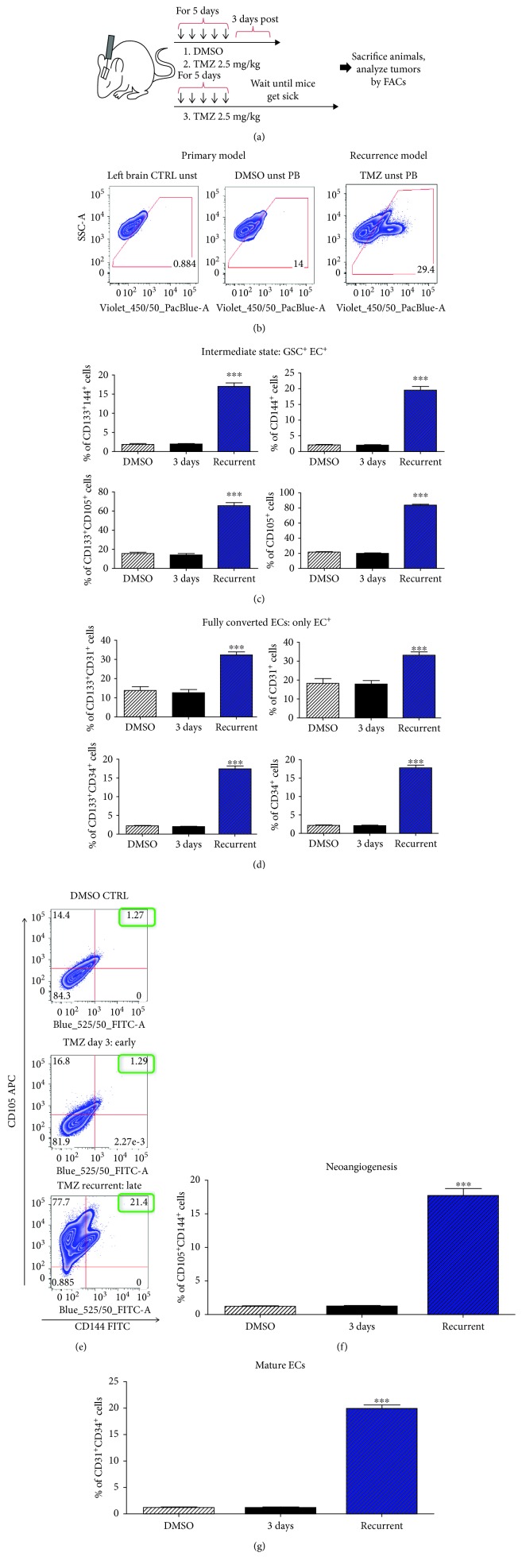
Recurrent GBM tumors exhibit increased expression of endothelial cell markers relative to primary tumors *in vivo*. (a) Schematic representation of experimental design. PDX GBM43 cells were implanted intracranially in the right cerebral hemisphere of athymic nude mice. Five days later, treatment was initiated, with three groups of mice receiving TMZ (2.5 mg) or equimolar DMSO. Mice were given the drug each day for five consecutive days and then were sacrificed three days following therapy. A second TMZ group was allowed to progress until tumor symptoms developed. Tumor cells were then analyzed by FACS. (b) Tumor cells were identified via use of a human-leukocyte antigen conjugated to Pacific Blue (PB). (c) Quantification of FACS analysis for percentage of cells positive for both CD133, a glioma stem cell marker, and CD105 or CD144, markers of intermediate endothelial cells. Note that these populations only reflect human cells. (d) Quantification of FACS analysis for percentage of cells positive for both CD133, a glioma stem cell marker, and CD34 or CD31, markers of mature endothelial cells. (e) Flow cytometry plots showing the expression of CD105 and CD144 in mice with implanted tumors treated with DMSO, mice treated with TMZ and sacrificed after 3 days, and mice treated with TMZ and sacrificed at the endpoint. (f, g). Quantification of flow cytometry showing a significant increase in the expression of immature EC markers CD105 and CD144 in mice treated with TMZ that developed recurrent brain tumors. The means of all mice were compared by one-way ANOVA with multiple comparisons. ^∗^ *p* < .05, ^∗∗^ *p* < .01, and ^∗∗∗^ *p* < .001. *N* = 5 mice/group.

These first mouse experiments dealt with what happens during the time period just after treatment with TMZ. However, the clinical course of GBM involves a longer period of time between administration of chemotherapy and the onset of lethal recurrence. In order to investigate the effects of TMZ on tumor-derived ECs and any temporal elements of this process, we turned to our primary and recurrent mouse models of GBM. Mice were injected with tumor cells as before and, after five days, treated with DMSO or TMZ (2.5 mg/kg). Some DMSO- and TMZ-treated mice were sacrificed after 3 days, while the remaining TMZ mice were sacrificed upon visible symptoms of tumor burden, thereby mimicking lethal recurrent tumors. Mice were euthanized and tumor cells analyzed by *ex vivo* FACS, using human leukocyte antigen (HLA) to identify human cells and avoid detection of murine blood vessels (Figures 5(a) and 5(b)). Analysis of CD133, CD105, and CD144 revealed that recurrent tumors have significantly increased subpopulations of tumor cells expressing both CD133 and an intermediate EC marker (Figure 5(c)). In addition, examination of CD31 and CD34 levels showed that TMZ also leads to the formation of tumor cells expressing mature EC markers (Figure 5(d)).

It is possible that some non-GSCs may also undergo transition to ECs or that some GSCs, in the process of conversion to an EC state, lose their CD133 expression. We therefore examined the expression of both immature and mature EC cells, regardless of CD133 expression. FACS analysis showed that TMZ treatment generates a subpopulation expressing both CD105 and CD144, suggesting ongoing conversion of tumor cells to ECs. It also showed a subpopulation of mature endothelial cells (Figures 5(e) and 5(f)).

**Figure 6:**
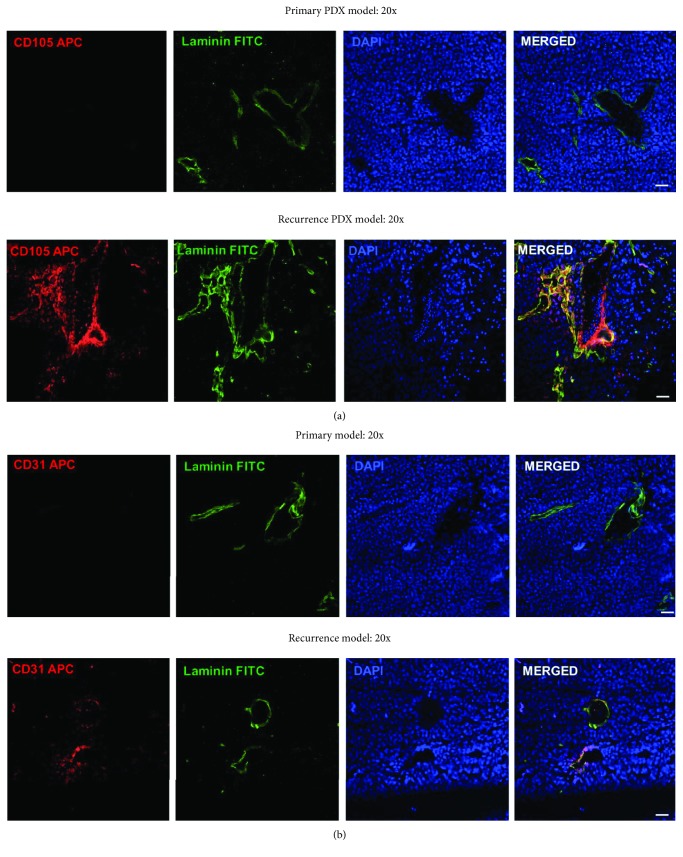
Recurrent PDX GBM43 tumors contain elevated tumor-derived vessels. Recurrent PDX GBM43 was established as described. (a) Brains containing tumors were removed and subjected to immunofluorescence (IF) analysis for CD105 with an antibody specific to the human antigen, a marker of intermediate endothelial cells and laminin, which indicates functional vessels. Tumors were identified via DAPI nuclear staining. (b) IF staining for CD31, a marker of mature endothelial cells and laminin. Images taken at 20x magnification.

Finally, to confirm that expression of these EC markers actually represents the formation of tumor-derived blood vessels, DMSO control tumors and recurrent TMZ-treated brains were stained for EC markers. This immunofluorescence showed that TMZ treatment does in fact increase expression of CD105 in tumor-derived blood vessels, as well as levels of Von Willebrand factor (VWF), a marker of functional vessels (Figure 6, Supplementary.  Figure 1). In all, these data strongly support our FACS analysis and show that TMZ treatment increases the formation of tumor-derived blood vessels.

## 4. Conclusions

Ongoing research continues to highlight GBM's ability to adapt to its environment as a key factor limiting the success of therapies. This study joins this chorus of studies and identifies a new connection between standard of care therapy and the generation of tumor-derived blood vessels. Our data demonstrate that (1) TMZ increases the expression of both GSC and EC markers and induces the formation of a subpopulation of GSC^+^EC^+^ cells, (2) these TMZ-induced endothelial cells generate tubes *in vitro*, (3) orthotopic xenografts also exhibit dose-dependent increases in the expression of EC markers following treatment, and (4) following treatment with TMZ, recurrent xenografts contain significantly more tumor-derived ECs and vessels. In sum, these results suggest that standard of care therapy promotes cellular plasticity leading to the generation of GBM-ECs and tumor-derived blood vessels. This process may represent a key portion of GBM's therapy response and contribute to lethal tumor recurrence.

These results confirm that GBM cells can not only attain a variety of tumor cell phenotypes but also transdifferentiate into tumor-supporting cells. In addition to endothelial cells [9, 10, 13], GBM cells can also become vascular pericytes [36]. Activation of this process by TMZ chemotherapy increases the number of tumor cells working towards the generation of new blood vessels.

In addition, our study offers a new perspective on the long-established role of hypoxia in GBM. Brain tumors, with their elevated VEGF levels and excessive vascularization, exhibit a high degree of regional microenvironmental heterogeneity, with hypoxic pockets [37, 38]. These areas are associated with GSCs and tumor aggression [5, 39]. Activation of hypoxia signaling has been shown to promote dedifferentiation during therapy [21, 40]. Critically, the work done in our lab has demonstrated that TMZ treatment increases the HIF response element expression in a recurrent model of glioma and this can lead to an acquisition of GSC status [21].

A key question surrounding the tumor-derived endothelium is the extent to which this process occurs. Our results suggest that up to 20% of tumor cells become mature endothelial cells. While not overwhelming, it is more than likely that this transdifferentiation acts in combination with tumor-activated neoangiogenesis to ensure ample blood flow.

Another critical question generated by these results is the potential mechanism governing this process. It has been shown that radiation causes increased secretion of VEGF [41]. Analysis of the patient's CSF confirmed that recurrent GBM tumors exhibited markedly elevated levels of VEGF, relative to primary GBM tumors [42]. This secretion has been linked to both neoangiogenesis and activation of the GSC state [3]. The therapeutic stress of radiation is known to induce secretion of VEGF in GBM [41], suggesting another possible connection. In addition, chemokine signaling by IL-8 has been linked to the induction of cellular plasticity and poor prognosis in GBM patients [43, 44]. Further research will delineate the role of specific signaling axes in vascular mimicry.

Finally, it remains an open question to what extent nontumor cells surrounding the tumor contribute to the generation of tumor-derived vessels. It is well established that endothelial and tumor cells participate in robust bidirectional communication [5, 6, 39]. Therefore, we speculate that signals from surrounding endothelial cells may contribute to the initiation of processes that generate tumor-derived vessels with one caveat. The core component of this study deals with GSCs and their response to TMZ treatment by inducing expression of vascular markers. We do not expect to see the mouse cell population contributing to GSC especially due to the difference in species; therefore, this effect can be discounted.

GBM tumors continue to show a remarkable ability to adapt to therapy. These results corroborate reports of this adaptation and highlight a potential new mechanism—formation of tumor-derived blood vessels. This result sheds light on the obstacles blocking the successful development of novel therapies against GBM.

## Data Availability

All data used to support the findings of this study are included within the article.

## Supplementary Materials

Supplementary Materials Figure S1: recurrent GBM tumors contain elevated tumor-derived vessels. (a) Brains containing tumors were removed and subjected to immunofluorescence (IF) analysis for CD105, a marker of intermediate endothelial cells and laminin, which indicates functional vessels. Tumors were identified via DAPI nuclear staining. (b) IF staining for VWF, a marker of mature blood vessels, and laminin. Images taken at 63x magnification. Figure S2: increasing doses of TMZ (50, 200, and 300 micromolar) alter CD133 expression levels as well as CD105 and CD31 expression in GSC population. (a) Representative FACS plots demonstrating CD133 APC fluorophore plotted against CD105 Pacific Blue Fluorophore. Cells treated with DMSO and TMZ are shown and quadrant percent is included. (b) Total percent of parent population of CD133 in DMSO and TMZ dosing conditions across days 2 and 4. (c) CD133 and CD105 double positive cells shown across DMSO and TMS doses for days 2 and 4. (d) CD133 and CD31 double positive cells shown across DMSO and TMZ doses for days 2 and 4. ^∗^ *p* < .05, ^∗∗^ *p* < .01, ^∗∗∗^ *p* < .001, and ^∗∗∗∗^ *p* < .0001.

## References

[1] Wick W., Osswald M., Wick A., Winkler F. (2018). Treatment of glioblastoma in adults. *Therapeutic Advances in Neurological Disorders*.

[2] Khasraw M., Ameratunga M. S., Grant R., Wheeler H., Pavlakis N., Cochrane Gynaecological, Neuro-oncology and Orphan Cancer Group (2014). Antiangiogenic therapy for high-grade glioma. *Cochrane Database of Systematic Reviews*.

[3] Hamerlik P., Lathia J. D., Rasmussen R. (2012). Autocrine VEGF–VEGFR2–neuropilin-1 signaling promotes glioma stem-like cell viability and tumor growth. *The Journal of Experimental Medicine*.

[4] Knizetova P., Ehrmann J., Hlobilkova A. (2008). Autocrine regulation of glioblastoma cell-cycle progression, viability and radioresistance through the VEGF-VEGFR2 (KDR) interplay. *Cell Cycle*.

[5] Calabrese C., Poppleton H., Kocak M. (2007). A perivascular niche for brain tumor stem cells. *Cancer Cell*.

[6] Charles N. A., Holland E. C. (2010). The perivascular niche microenvironment in brain tumor progression. *Cell Cycle*.

[7] Liu D., Martin V., Fueyo J. (2010). Tie2/TEK Modulates the Interaction of Glioma and Brain Tumor Stem Cells with Endothelial Cells and Promotes an Invasive Phenotype. *Oncotarget*.

[8] Cuddapah V. A., Robel S., Watkins S., Sontheimer H. (2014). A neurocentric perspective on glioma invasion. *Nature Reviews Neuroscience*.

[9] Ricci-Vitiani L., Pallini R., Biffoni M. (2010). Tumour vascularization via endothelial differentiation of glioblastoma stem-like cells. *Nature*.

[10] Scully S., Francescone R., Faibish M. (2012). Transdifferentiation of glioblastoma stem-like cells into mural cells drives vasculogenic mimicry in glioblastomas. *Journal of Neuroscience*.

[11] Francescone R., Scully S., Bentley B. (2012). Glioblastoma-derived tumor cells induce vasculogenic mimicry through Flk-1 protein activation. *Journal of Biological Chemistry*.

[12] Chiao M. T., Yang Y. C., Cheng W. Y., Shen C. C., Ko J. L. (2011). CD133+ glioblastoma stem-like cells induce vascular mimicry in vivo. *Current Neurovascular Research*.

[13] Wang R., Chadalavada K., Wilshire J. (2010). Glioblastoma stem-like cells give rise to tumour endothelium. *Nature*.

[14] Liu X. M., Zhang Q. P., Mu Y. G. (2011). Clinical significance of vasculogenic mimicry in human gliomas. *Journal of Neuro-Oncology*.

[15] Liau B. B., Sievers C., Donohue L. K. (2017). Adaptive chromatin remodeling drives glioblastoma stem cell plasticity and drug tolerance. *Cell Stem Cell*.

[16] Safa A. R., Saadatzadeh M. R., Cohen-Gadol A. A., Pollok K. E., Bijangi-Vishehsaraei K. (2015). Glioblastoma stem cells (GSCs) epigenetic plasticity and interconversion between differentiated non-GSCs and GSCs. *Genes & Diseases*.

[17] Heddleston J. M., Li Z., McLendon R. E., Hjelmeland A. B., Rich J. N. (2009). The hypoxic microenvironment maintains glioblastoma stem cells and promotes reprogramming towards a cancer stem cell phenotype. *Cell Cycle*.

[18] Hu Y. L., DeLay M., Jahangiri A. (2012). Hypoxia-induced autophagy promotes tumor cell survival and adaptation to antiangiogenic treatment in glioblastoma. *Cancer Research*.

[19] Soeda A., Park M., Lee D. (2009). Hypoxia promotes expansion of the CD133-positive glioma stem cells through activation of HIF-1alpha. *Oncogene*.

[20] Evans S. M., Judy K. D., Dunphy I. (2004). Hypoxia is important in the biology and aggression of human glial brain tumors. *Clinical Cancer Research*.

[21] Lee G., Auffinger B., Guo D. (2016). Dedifferentiation of glioma cells to glioma stem-like cells by therapeutic stress-induced HIF signaling in the recurrent GBM model. *Molecular Cancer Therapeutics*.

[22] Bruns A. F., Bao L., Walker J. H., Ponnambalam S. (2009). VEGF-A-stimulated signalling in endothelial cells via a dual receptor tyrosine kinase system is dependent on co-ordinated trafficking and proteolysis. *Biochemical Society Transactions*.

[23] Sun B., Zhang D., Zhang S., Zhang W., Guo H., Zhao X. (2007). Hypoxia influences vasculogenic mimicry channel formation and tumor invasion-related protein expression in melanoma. *Cancer letters*.

[24] De Palma M., Biziato D., Petrova T. V. (2017). Microenvironmental regulation of tumour angiogenesis. *Nature Reviews Cancer*.

[25] Hodgson J. G., Yeh R. F., Ray A. (2009). Comparative analyses of gene copy number and mRNA expression in glioblastoma multiforme tumors and xenografts. *Neuro-Oncology*.

[26] Auffinger B., Tobias A. L., Han Y. (2014). Conversion of differentiated cancer cells into cancer stem-like cells in a glioblastoma model after primary chemotherapy. *Cell Death & Differentiation*.

[27] Beier D., Rohrl S., Pillai D. R. (2008). Temozolomide preferentially depletes cancer stem cells in glioblastoma. *Cancer Research*.

[28] Zhang Y., Liu T., Meyer C. A. (2008). Model-based analysis of ChIP-Seq (MACS). *Genome Biology*.

[29] Brada M., Judson I., Beale P. (1999). Phase I dose-escalation and pharmacokinetic study of temozolomide (SCH 52365) for refractory or relapsing malignancies. *British Journal of Cancer*.

[30] Rosso L., Brock C. S., Gallo J. M. (2009). A new model for prediction of drug distribution in tumor and normal tissues: pharmacokinetics of temozolomide in glioma patients. *Cancer Research*.

[31] Smith S. J., Tilly H., Ward J. H. (2012). CD105 (endoglin) exerts prognostic effects via its role in the microvascular niche of paediatric high grade glioma. *Acta Neuropathologica*.

[32] Lee C. H., Wu Y. T., Hsieh H. C., Yu Y., Yu A. L., Chang W. W. (2014). Epidermal growth factor/heat shock protein 27 pathway regulates vasculogenic mimicry activity of breast cancer stem/progenitor cells. *Biochimie*.

[33] Pisacane A. M., Picciotto F., Risio M. (2007). CD31 and CD34 expression as immunohistochemical markers of endothelial transdifferentiation in human cutaneous melanoma. *Cellular Oncology*.

[34] Patel A. P., Tirosh I., Trombetta J. J. (2014). Single-cell RNA-seq highlights intratumoral heterogeneity in primary glioblastoma. *Science*.

[35] Verhaak R. G., Hoadley K. A., Purdom E. (2010). Integrated genomic analysis identifies clinically relevant subtypes of glioblastoma characterized by abnormalities in PDGFRA, IDH1, EGFR, and NF1. *Cancer Cell*.

[36] Cheng L., Huang Z., Zhou W. (2013). Glioblastoma stem cells generate vascular pericytes to support vessel function and tumor growth. *Cell*.

[37] Spence A. M., Muzi M., Swanson K. R. (2008). Regional hypoxia in glioblastoma multiforme quantified with [18F]fluoromisonidazole positron emission tomography before radiotherapy: correlation with time to progression and survival. *Clinical Cancer Research*.

[38] Evans S. M., Judy K. D., Dunphy I. (2004). Comparative measurements of hypoxia in human brain tumors using needle electrodes and EF5 binding. *Cancer Research*.

[39] Gilbertson R. J., Rich J. N. (2007). Making a tumour’s bed: glioblastoma stem cells and the vascular niche. *Nature Reviews Cancer*.

[40] Dahan P., Martinez Gala J., Delmas C. (2014). Ionizing radiations sustain glioblastoma cell dedifferentiation to a stem-like phenotype through survivin: possible involvement in radioresistance. *Cell Death & Disease*.

[41] Hovinga K. E., Stalpers L. J. A., van Bree C. (2005). Radiation-enhanced vascular endothelial growth factor (VEGF) secretion in glioblastoma multiforme cell lines--a clue to radioresistance?. *Journal of Neuro-Oncology*.

[42] Salmaggi A., Eoli M., Frigerio S. (2003). Intracavitary VEGF, bFGF, IL-8, IL-12 levels in primary and recurrent malignant glioma. *Journal of Neuro-Oncology*.

[43] Sharma I., Singh A., Siraj F., Saxena S. (2018). IL-8/CXCR1/2 signalling promotes tumor cell proliferation, invasion and vascular mimicry in glioblastoma. *Journal of Biomedical Science*.

[44] Angara K., Borin T. F., Rashid M. H. (2018). CXCR2-expressing tumor cells drive vascular mimicry in antiangiogenic therapy-resistant glioblastoma. *Neoplasia*.

