# Supplementary material for: Chemotherapeutic Stress Induces Transdifferentiation of Glioblastoma Cells to Endothelial Cells and Promotes Vascular Mimicry
Source: Stem Cells Int. 2019 Jun 18;2019:6107456. doi: 10.1155/2019/6107456 (PMC6604352; doi:10.1155/2019/6107456)
Supplement: Supplementary Materials — Figure S1: recurrent GBM tumors contain elevated tumor-derived vessels. (a) Brains containing tumors were removed and subjected to immunofluorescence (IF) analysis for CD105, a marker of intermediate endothelial cells and laminin, which indicates functional vessels. Tumors were identified via DAPI nuclear staining. (b) IF staining for VWF, a marker of mature blood vessels, and laminin. Images taken at 63x magnification. Figure S2: increasing doses of TMZ (50, 200, and 300 micromolar) alter CD133 expression levels as well as CD105 and CD31 expression in GSC population. (a) Representative FACS plots demonstrating CD133 APC fluorophore plotted against CD105 Pacific Blue Fluorophore. Cells treated with DMSO and TMZ are shown and quadrant percent is included. (b) Total percent of parent population of CD133 in DMSO and TMZ dosing conditions across days 2 and 4. (c) CD133 and CD105 double positive cells shown across DMSO and TMS doses for days 2 and 4. (d) CD133 and CD31 double positive cells shown across DMSO and TMZ doses for days 2 and 4. ∗ p < .05, ∗∗ p < .01, ∗∗∗ p < .001, and ∗∗∗∗ p < .0001. [file 6107456.f1.zip › 6107456.f1/TD_FigS1_SCI_2747063.pptx]

## Slide 1
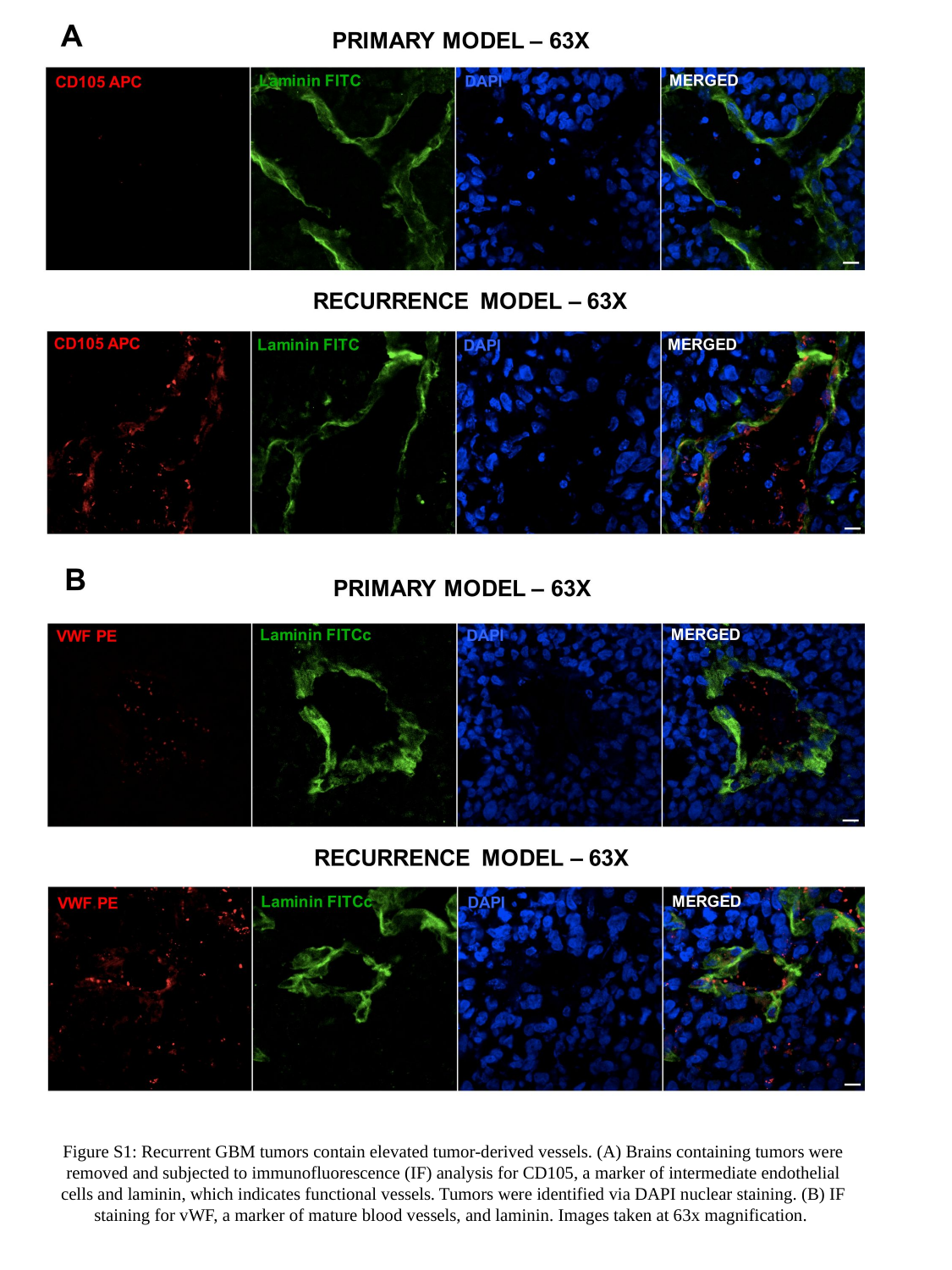

Figure S1: Recurrent GBM tumors contain elevated tumor-derived vessels. (A) Brains containing tumors were removed and subjected to immunofluorescence (IF) analysis for CD105, a marker of intermediate endothelial cells and laminin, which indicates functional vessels. Tumors were identified via DAPI nuclear staining. (B) IF staining for vWF, a marker of mature blood vessels, and laminin. Images taken at 63x magnification.
